# A real-time RT-PCR for detection of clade 1 and 2 H5N1 Influenza A virus using Locked Nucleic Acid (LNA) TaqMan probes

**DOI:** 10.1186/1743-422X-7-46

**Published:** 2010-02-22

**Authors:** Thanh Tran Tan, Hana Apsari Pawestri, Ngoc Nghiem My, Hien Vo Minh, Harun Syahrial, Trung Nguyen Vu, H Rogier van Doorn, Heiman FL Wertheim, Chau Nguyen Van Vinh, Ha Do Quang, Jeremy J Farrar, Hien Tran Tinh, Endang R Sedyaningsih, Menno D de Jong

**Affiliations:** 1 Oxford University Clinical Research Unit, Hospital for Tropical Diseases, 190 Ben Ham Tu, Dist 05, Ho Chi Minh City, Viet Nam; 2 National Institute of Health Research and Development, Percetakan Negara no. 29, Jakarta 10560, Indonesia; 3 Hospital for Tropical Diseases, 190 Ben Ham Tu, Dist 05, Ho Chi Minh City, Viet Nam; 4 National Institute of Infectious and Tropical Diseases, Ha Noi, Viet Nam; 5 Oxford University Clinical Research Unit - The National Institute of Infectious and Tropical Diseases, Ha Noi, Viet Nam; 6 Centre for Tropical Medicine, Nuffield Department of Clinical Medicine, University of Oxford, Centre for Clinical Vaccinology and Tropical Medicine, Oxford, UK; 7 Department of Medical Microbiology, Academic Medical Center, University of Amsterdam, Amsterdam, The Netherlands

## Abstract

**Background:**

The emergence and co-circulation of two different clades (clade 1 and 2) of H5N1 influenza viruses in Vietnam necessitates the availability of a diagnostic assay that can detect both variants.

**Results:**

We developed a single real-time RT-PCR assay for detection of both clades of H5N1 viruses, directly from clinical specimens, using locked nucleic acid TaqMan probes. Primers and probe used in this assay were designed based on a highly conserved region in the *HA *gene of H5N1 viruses. The analytical sensitivity of the assay was < 0.5 PFU and 10 - 100 ssDNA plasmid copies. A total of 106 clinical samples (58 from patients infected with clade 1, 2.1 or 2.3 H5N1 viruses and 48 from uninfected or seasonal influenza A virus-infected individuals) were tested by the assay. The assay showed 97% concordance with initial diagnostics for H5 influenza virus infection with a specificity of 100%.

**Conclusions:**

This assay is a useful tool for diagnosis of H5N1 virus infections in regions where different genetic clades are co-circulating.

## Background

Highly pathogenic avian influenza A (H5N1) viruses cause sporadic infections in humans, and are associated with severe respiratory disease with a mortality of about 60% [1]. Since the re-emergence of human H5N1 influenza virus infections in January 2003 [2], 436 human cases have been documented in 15 countries in Asia, Africa, and Europe [1]. Genetic studies have revealed that most of the viruses isolated from humans and poultry belong to genotype Z [3,4]. The worldwide distribution of this genotype has resulted in the establishment of at least two genetically and geographically distinct clades: clade 1 and 2 [5]. Clade 1 H5N1 viruses have been isolated from poultry and humans in Vietnam, Thailand, and Cambodia, and from poultry in Laos and Malaysia [6-8]. Clade 2 viruses have a larger genetic diversity and are divided into 5 sub-clades (2.1 to 2.5) [9]. Clade 2.1 viruses have been found only in Indonesia, in poultry and humans [6]. Clade 2.2 viruses have caused poultry outbreaks and human infections in the Middle East, Africa, and Europe [1]. Clade 2.3 viruses are further divided into four sub-clades (2.3.1 to 2.3.4) [9]. Recently, clade 2.3.4 viruses have become predominant in China and have also been reported in Hong Kong, Laos, Malaysia, Thailand, and North-Vietnam [10,11]. In Vietnam, clades 1 and 2.3.4 co-circulate among poultry and have both caused human infections [11,12].

The circulation of more than one virus clade poses a challenge for laboratory diagnostics, since methods for detection of H5N1 infection usually rely on clade specific amplification of the *HA *gene [13-15]. Although rapid antigen tests, virus isolation, and serological tests can be used to diagnose H5N1 infection across all clades, these methods have limited use for routine diagnostics because of the inability to subtype, the low sensitivity, and the requirement of biosafety level 3 laboratory facilities. The accepted reference method for diagnosis of H5N1 infection is real-time RT-PCR (rRT-PCR) [16]. Compared to conventional RT-PCR, rRT-PCR has a smaller risk of cross-contamination, higher sensitivity and specificity, and shorter per sample laboratory turnaround time. Several rRT-PCR assays for H5N1 detection have been described [15,17-20], but only two of them have been specifically designed for the detection of both clades [19,20]. In addition, clinical evaluation has not been performed for most of these assays [15,18-20].

Recently, the locked nucleic acid (LNA) technology has been integrated into real-time PCR, enabling a more flexible primer and probe design and improving amplification efficiency [21-23]. In this study, we describe the use and evaluation of an LNA TaqMan rRT-PCR for detection of clade 1 and 2 H5N1 viruses in a large number of clinical specimens (n = 58).

The assay described here has been established within the laboratories of the South East Asia Infectious Disease Clinical Research Network [24] to serve as a supplementary diagnostic test in addition to the FDA - approved USCDC assay [25] for Influenza virus infection and H5N1 subtyping.

## Results

### Analytical sensitivity and specificity

The analytical sensitivity of our LNA Taqman rRT-PCR for the detection of the *HA *gene of H5N1 was < 0.5 PFU of virus and 10 copies of ssDNA plasmids. No fluorescence was detected when analyzing human seasonal H1N1 (n = 4) and H3N2 (n = 5) virus isolates and non-H5 avian viruses (n = 5), indicating a high specificity for influenza A viruses of subtype H5.

### Evaluation of sensitivity and specificity in clinical specimens

The sensitivity of the assay was clinically evaluated in 58 human specimens, previously confirmed to contain clade 1, clade 2.1, or clade 2.3 H5N1 virus by virus isolation and/or H5N1 specific RT-PCRs [25,26] and sequencing (our unpublished data). Our assay detected H5 virus in 56 of these samples (97%). The sensitivity was 100% for clade 1 and clade 2.3, and 92% for clade 2.1 (Table 1).

**Table 1:** H5N1 clinical samples and rRT-PCR results

Samples/virus clade	NS	TS	TA	Plas	PF	Stool	Total	rRT-PCR positive
Clade 1	2	7	1	0	0	0	10	10

Clade 2.1	7	17	1	0	0	0	25	23

Clade 2.3	5	7	6	2	2	1	23	23

Total	14	31	8	2	2	1	58	56

rRT-PCR positive	13	30	8	2	2	1	56	

NS = Nasal swab; TS = Throat swab; TA = Tracheal aspirate; Plas = Plasma; PF = Pleural fluid.

The specificity of the assay in clinical specimens was assessed by analyzing influenza A H1 or H3 positive samples (n = 19) and influenza negative (n = 29) respiratory samples. All of these samples were negative indicating 100% specificity.

## Discussion

Recent evidence of co-circulation of clade 1 and clade 2 H5N1 viruses in South East Asia has highlighted the need for RT-PCR assays that allow detection of both genetic clades. We developed a single step rRT-PCR assay using an LNA TaqMan probe for direct detection in clinical samples of *HA *genes from both clades of H5N1 viruses. This assay was shown to be sensitive, specific, and rapid (approximately 3.5 hours after RNA extraction).

The primers and probe used in this study were designed to target a highly conserved region in the *HA *gene of H5N1 viruses; to ensure amplification of both clade 1 and 2 RNA, one and two degenerated bases were incorporated into the forward and reverse primers, respectively. As the binding efficiency of the original TaqMan probe was inadequate, LNA residues were incorporated that result in a tighter crosslink than normal nucleotides. Incorporation of LNA residues has no consequences for PCR conditions [27] but is a practical way to improve probe-binding efficiency [28].

The assay was shown to be sensitive, detecting 10 copies of ssDNA plasmid, and less than 0.5 PFU of H5N1 viruses per reaction, and specific for the detection of influenza A of subtype H5. The *HA *gene of clades 1, 2.1, and 2.3 was amplified from both virus isolates and human clinical specimens. Cross-reaction with virus isolates from other influenza A subtypes was not observed, and no positive results were obtained when analyzing 48 clinical samples from patients with either seasonal influenza or non-influenza respiratory illness.

Clinical evaluation was performed on 58 stored clinical specimens from 39 patients infected with either clade 1, 2.1 or 2.3 viruses and showed high concordance when compared to initial diagnostic RT-PCR and/or cell culture results. To our knowledge, the number of H5 positive clinical specimens used in this study is larger than in any other previously published assays [17].

Our assay has not been evaluated in clade 2.2 H5N1 viruses and clinical specimens. However, in silico analysis of clade 2.2 viruses showed that the primers and probe used this assay would hybridize sufficiently with viruses of this sub-clade to allow amplification (data not shown).

Our assay failed to detect virus in a nasal swab and a throat swab (Table 1). This may be due to RNA degradation during long-term storage and multiple freeze-thaw cycles.

## Conclusions

We have developed a highly sensitive and specific rRT-PCR assay for the detection of H5N1 influenza A virus of both clade 1 and 2 directly in clinical specimens, and evaluated it with a large number clinical samples. Using this assay, reliable diagnostic results can be obtained in a few hours, thus enabling timely clinical management and outbreak control.

## Methods

### Cell-lines and isolates

For sensitivity and specificity analyses, the following virus isolates were used: 12 clade 1 human H5N1 viruses, isolated from patients admitted to the Hospital for Tropical Diseases, Ho Chi Minh City, Viet Nam in 2004 and 2005 [29]; 4 clade 2.3.4 H5N1 viruses isolated from patients admitted to the National Institute of Infectious and Tropical Diseases, Ha Noi, Vietnam in 2007 and 2008; 1 human clade 2.1 H5N1 isolate (A/Indonesia/5/2005(H5N1)), kindly provided by The National Institute of Infectious Diseases, Tokyo, Japan; 9 human influenza A viruses of subtype H1N1 (n = 4) and H3N2 (n = 5), isolated from patients with seasonal influenza from Dong Thap Province, Vietnam, in 2006; and 7 avian influenza viruses of subtypes H3 (n = 1), H4 (n = 3), H5 (n = 2), and H6 (n = 1), isolated from poultry in 2006 in Ho Chi Minh City, and the southern Vietnamese provinces of Vinh Long, and Dong Thap.

All viruses were cultured in Madin Darby Canine Kidney cells (ECACC, Wiltshire, UK) and were subtyped using previously described methods [26,30].

### Clinical samples

Fifty-eight clinical samples from 39 H5N1-infected patients were used in this study (Table 1), including nasal swabs (n = 14), throat swabs (n = 31), nasopharyngeal aspirates (n = 8), stools (n = 1), plasma (n = 2), and pleural fluids (n = 2). Swabs were collected in viral transport medium and stored at -80°C. Initial diagnoses in these patients were made independently in Jakarta, Ha Noi, and Ho Chi Minh City by RT-PCR and/or virus isolation using previously described methods [26,25]. The samples were collected from patients with H5N1 infection in Indonesia (clade 2.1; 25 specimens from 25 patients) and Vietnam (clade 1; 10 specimens from 10 patients; clade 2.3.4: 23 specimens from 4 patients) between 2004 and 2008 (Table 1).

Nineteen throat swab samples from 19 patients with seasonal influenza (seasonal H1N1: n = 10; H3N2: n = 9), confirmed by conventional RT-PCRs and/or virus isolation as described previously [26], and 29 throat swab samples from 29 patients with non-influenza respiratory illness admitted to the Hospital for Tropical Diseases Ho Chi Minh City during the H5N1 outbreaks of 2004 - 2005 were also used in this study.

All laboratory analyses in specimens from Indonesian patients for this study were performed at the National Institute of Health Research and Development, Ministry of Health, Jakarta, Indonesia; analyses of specimens from Vietnamese patients were done at the Hospital for Tropical Diseases, Ho Chi Minh City, Vietnam.

### Ethical approval

Clinical specimens from H5N1 patients in Ho Chi Minh City and negative control specimens were obtained as part of studies on H5N1 and respiratory infections that were approved by the institutional review board of the Hospital for Tropical Diseases, Ho Chi Minh City, and the Oxford Tropical Research Ethical Committee. Informed consent was obtained from all participating patients or their parents or legal guardians.

The clinical specimens from Indonesian patients and from patients from Ha Noi were obtained by health care providers from suspected H5N1 cases as part of the national procedures for Avian Influenza case investigation which were exempted from review by the institutional review boards.

### RNA extraction

Viral RNA was extracted from 140 μl of clinical samples or from 50 μl of culture supernatant and eluted in 60 μl elution buffer using QIAamp Viral RNA Mini kit (Qiagen, West Sussex, UK) according to the manufacturer's instructions.

### Primer and probe design

Nucleotide sequences of all available full length *HA *genes of H5N1 viruses (N = 313) were retrieved from Influenza Virus Resource [31] and were aligned using BioEdit version 7.0.1 (Ibis Biosciences, Carlsbad, CA, USA). Primers and probe were designed using Primer Express version 2.0 (Applied Biosystems Inc., Foster City, CA, USA). The probe was further modified using LNA residues. Primers and probes (Table 2) were synthesized by Sigma-Proligo, Singapore.

**Table 2:** Primers and probe used in this study

Name	Sequence^a^	Nucleotide^b^
Sense	5'-TTGGTTACCATGCAAACAAYT-3'	91-111

Antisense	5'-TRTCTTGGGCRTGTGTAACA-3	152-171

Probe	5'-FAM-CAGGTTG**A**C**A**CA**A**TA**A**TGGAAA**A**G-BHQ3-3'	119-143

^a ^Y = T or C, R = A or G. LNA residues in the probe are indicated in bold. 5' - FAM = 5' - carboxyfluorescein, BHQ = Black Hole Quencher. ^b ^The position in the *HA *gene is indicated.

### Determination of sensitivity

For determination of analytical sensitivity, a representative clade 1 (strain A/Vietnam/CL115/2005(H5N1)) was titrated in MDCK cells, and serial dilutions at concentrations of 10^4 ^- 10^-2 ^PFU/μl were made. From each resulting dilution, viral RNA was extracted and subjected to rRT-PCR.

In addition, sensitivity analyses were performed as follows: PCR products from amplification of the *HA *gene of A/Vietnam/CL115/2005(H5N1) were purified using the QIAquick^® ^PCR purification kit (Qiagen), and were cloned into pCR2.1-TOPO plasmid, and were then used for transformation of *E. coli *TOP 10 cells (Invitrogen, Carlsbad, CA, USA). The plasmids derived from a single bacterial colony were sequenced using CEQ Dye Terminator Cycle Sequencing Kit (Beckman Coulter, Fullerton, CA, USA). Selected clones were propagated in liquid LB medium according to the manufacturer's instructions and plasmids were purified using QIAprep^® ^Miniprep Kit (Qiagen). After linearization using *Xho*I (New England Biolabs, Ipswich, UK), DNA concentration was determined spectrophotometrically (NanoDrop 1000, Thermo Scientific, Wilmington, DE, USA). The plasmids were then diluted tenfold in TE (10^5 ^- 10^-2 ^copies/μl) and were used in analytical sensitivity tests.

All experiments were done in duplicate.

### Real-time RT-PCR

Real-time RT-PCR was performed using iScript™ One-Step RT-PCR Kit Probes in Chromo 4 real time PCR machines (Bio-Rad, Hercules, CA, USA). The reaction was conducted in a total volume of 25 μl containing 12.5 μl of 2× RT-PCR Reaction Mix, 400 nM of each primer, 120 nM of probe, 0.5 μl of iScript Reverse Transcriptase, and 5 μl of template. Optimized rRT-PCR conditions were as follows: one cycle of 50°C for 15 minutes, followed by 5 minutes at 95°C, and 45 cycles of 15 seconds at 95°C and 1 minute at 53°C.

## Competing interests

This work was supported by the South East Asia Infectious Diseases Clinical Research Network. We have no competing interests.

## Authors' contributions

TTT: designed the study, did laboratory testing, analysed the test results, and drafted the manuscript.

HAP, NNM, HVM, SH, TNV, HFLW, CNVV, HDQ, and HTT: enrolled patients, took samples and did laboratory testing.

RHvD, JJF, ERS, and MDdJ: designed the study and were involved in drafting the manuscript.

All authors have read the final manuscript and agreed with its contents.
